# Evaluation of the Factors Affecting the Cure Rate of Cervical Intra-Epithelial Neoplasia Recurrence Using Defective Models

**DOI:** 10.34172/jrhs.2021.56

**Published:** 2021-07-12

**Authors:** Nastaran Hajizadeh, Ahmad Reza Baghestani, Mohamad Amin Pourhoseingholi, Ali Akbar Khadem Maboudi, Farah Farzaneh, Nafiseh Faghih

**Affiliations:** ^1^Department of Biostatistics, Faculty of Paramedical Sciences, Shahid Beheshti University of Medical Sciences, Tehran, Iran; ^2^Physiotherapy Research Center, Department of Biostatistics, Faculty of Paramedical Sciences, Shahid Beheshti University of Medical Sciences, Tehran, Iran; ^3^Gastroenterology and Liver Diseases Research Center, Research Institute for Gastroenterology and Liver Diseases, Shahid Beheshti University of Medical Sciences, Tehran, Iran; ^4^Department of Biostatistics, Faculty of Paramedical Sciences, Shahid Beheshti University of Medical Sciences, Tehran, Iran; ^5^Preventive Gynaecology Research Center, Taleghani Hospital, Shahid Beheshti University of Medical Sciences, Tehran, Iran

**Keywords:** Cervical intra-epithelial neoplasia, Cure rate, Defective models, Survival analysis

## Abstract

**Background:** Treatment of cervical intraepithelial neoplasia is very important since if it remains untreated, it may progress to cervical cancer. It is usually treated with excisional surgery. This study aimed to find the factors affecting the cure rate of cervical intraepithelial neoplasia recurrence after surgery using defective models.

**Study design:** A retrospective cohort study.

**Methods:** Excisional surgery was performed on 307 patients with high-grade cervical intraepithelial neoplasia, from 2009 to 2017. The patients were followed up until recurrence based on histopathology report. Hematologic factors were measured before surgery. The cure rates were estimated using defective models with a Gamma frailty term and the results were compared.

**Results:** Neutrophil-to-lymphocyte ratio (NLR) (*P*<0.001) and excised mass size (*P*<0.001) had significant impacts on cure rates, and their cut-off values were 1.9 (*P*<0.001) and 15 mm^2^ (*P*<0.001), respectively. Patients with lower neutrophil-to-lymphocyte ratios and larger excised tissues had higher cure rates. Defective 3-parameter Gompertz distribution with gamma frailty term had the best fit to the data, and its estimated cure rates were 98% among patients with an excised mass size of >15 mm^2^ and NLR of <1.9, 84% among patients with an excised mass size of >15 mm^2^ and NLR of >1.9, 79% among patients with an excised mass size of <15 mm^2^ and NLR of <1.9, and 30% among patients with an excised mass size of <15 mm^2^ and NLR of >1.9.

**Conclusion:** Cervical intraepithelial neoplasia must be identified and treated before its progress. Excision of more tissues during excisional surgery, especially when the NLR of the patient is high, can help to prevent cervical intraepithelial neoplasia recurrence.

## Introduction

Cervical cancer is the third most frequent cancer among women, with an age-standardized incidence rate of 13.1 per 100,000 and an age-standardized mortality rate of 6.9 per 100,000 in the world^1^. The pre-invasive cervical lesion is a type of detectable epithelial change which if left untreated, may progress to an advanced form of cervical cancer^2^. Therefore, detection and treatment of cervical intraepithelial neoplasia (CIN) as a pre-invasive lesion can prevent cervical cancer^3-7^.

Generally, CIN is divided into three grades: (i) CIN I which is equivalent to mild dysplasia, (ii) CIN II which is equivalent to moderate dysplasia, and (iii) CIN III which is equivalent to severe dysplasia or in-situ carcinoma and recognized as a true pre-invasive precursor with a potential to progress to cancer^8,9^. The CIN II and III are often treated with one of the local excisional procedures which has proved to be effective^10^. The most common CIN excisional procedures are large loop excision of the transformation zone, loop electrosurgical excision procedure, laser conization, and cold-knife conization^11,12^.

There are several factors that could play signiﬁcant roles in the prediction of CIN recurrence rate, such as age, marginal involvement of the sample, glandular involvement, chronic inflammation caused by bacterial or viral infections, and the status of the immune system of the body^13-15^. There are various factors for checking the status of the immune system. Among them, neutrophil–to–lymphocyte ratio (NLR) is an effective marker of inflammation and is calculated by dividing the absolute peripheral blood neutrophils count by the absolute lymphocytes count^16^.

**Figure 1:**
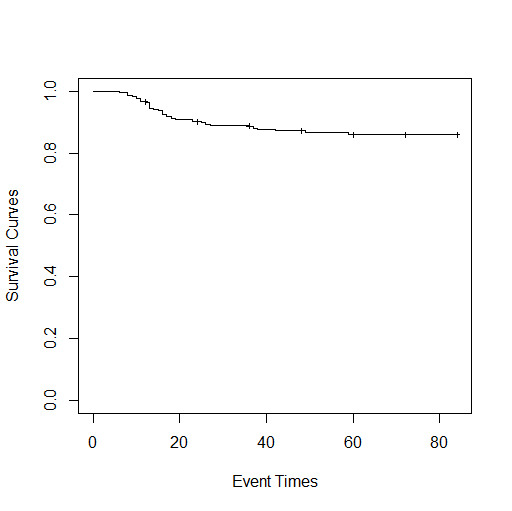
Kaplan-Meier curve of the cervical intraepithelial neoplastic data

Standard survival models assume that all subjects are susceptible to the event of interest (such as recurrence or death from the disease)^17,18^. However, in practice, some individuals will never experience the event of interest; these risk-free subjects are called ‘‘cured’’. Existence of the cured fraction is indicated by a long ﬂat tail which is not close to zero in Kaplan-Meier curves^19^ as it is shown in Figure 1.

The mixture models are usually used for cure rate modeling. The survival function of the standard mixture model is S(t)=p+(1-p)S_0_(t), where p∈(0,1) and S_0_(t) is a usual survival function that converges to zero as time goes to infinity. Therefore, S(t) converges to p with the passage of time^17^. Recently, defective distributions are used for cure rate modeling^20^. Defective distributions have the ability to become a cure rate model by changing the usual domain of its parameters without adding any extra parameters to the model. It should be mentioned that the parameters whose domains change are called defective parameters^20^. The proportion of the cured population is acquired by calculating the limit of the defective survival function which is a value between zero and one^21-25^. There are two defective distributions in the related literature, namely the Gompertz and the Inverse Gaussian distributions.

It is known that two subjects with the same observed characteristics may have different survival times due to the factors that are not or could not be observed among people, such as genetic and environmental factors. Frailty models accommodate the unobserved heterogeneity by the inclusion of a random effect in the model which is called an individual frailty term and improves the fit of the model. The gamma distribution is commonly used for the frailty term^26^.

 The present study aimed to estimate the cured fraction and identify the factors affecting the cure rate of CIN recurrence among 307 women treated with CIN excisional surgery using defective models with frailty terms.

## Methods

The required data were collected from a historical cohort study performed on 307 patients with CIN-positive pathology. They had excisional treatment at the Department of Oncology, Imam Hossein Hospital, Tehran, Iran from 2009 to 2017 and were followed up until January 2018^27^. It should be mentioned that during this period, 514 individuals were treated, while 14 of them were excluded due to impairment of immunity and underlying illnesses, and 193 of them were excluded due to the lack of follow-up. During this period, individuals with high-grade CIN (II and III) were treated by loop electrosurgical excision procedure or cold-knife conization excisional procedures and were followed up with performing colposcopy at 6 and 12 months, and then annually.

 The recurrence was determined by histopathology report, and the time interval between surgery and recurrence was considered the survival time. The survival time is right-censored for patients without recurrence. Also, in the case of hysterectomy, the patients were usually cured, therefore, their survival time was considered as right-censored. Demographic, clinical, pathological, and hematological findings of these patients were extracted from their records. Moreover, complete blood count tests were taken before surgery and the NLR and platelet–to–lymphocyte ratio indexes were calculated using neutrophil, platelet, and lymphocyte counts reported in the blood test.

 Regarding ethical considerations, written informed consent was obtained from patients and the study was approved by the Ethics Committee of Shahid Beheshti University of Medical Sciences, Tehran, Iran (IR.SBMU.RETECH. 1397.1360).

###  Statistical analysis

Mean and standard deviation values were presented for quantitative variables and evaluated based on one-way ANOVA test. Frequency and percentage were presented for qualitative variables and evaluated using the Chi-square test. Effects of different variables on the cure rate were assessed using two defective models with a frailty term: the defective Gompertz model with Gamma frailty term (defective Gamma Gompertz model), and the defective 3-parameter Gompertz model with Gamma frailty term (defective Gamma 3-parameter Gompertz model)^28,29^. It should be mentioned that the inverse Gaussian model with gamma frailty term was also fitted to the data but was not able to estimate the cure rate in this dataset. This is why this model is not explained here.

 The survival function of the Gompertz distribution with gamma frailty term is as follows:


(1) $$S\left(t\left|x\right.\right)={\left\{1+\theta \frac{e^{x^{T}\beta }}{\alpha }\left(e^{\alpha t}-1\right)\right\}}^{-\frac{1}{\theta }}$$

Here, α>0 represents the shape parameter, θ>0 is the frailty term, and x^T^β=b0+b_1_x_1_+b_2_x_2_+…b_k_x_k_ in which β=(b_0_,b_1_,…, b_k_) indicates the coefficients vector andx^T^=(1,x_1_, x_2_,…, x_k_) indicates the covariates vector. When α<0, we have the defective gamma-Gompertz (DGG) model and θ∈R.

 The hazard function of the DGG model is as follows:


(2) $$h\left(t\left|x\right.\right)=e^{\alpha t+x^{T}\beta }{\left\{1+\theta \frac{e^{x^{T}\beta }}{\alpha }\left(e^{\alpha t}-1\right)\right\}}^{-1}$$

The cure fraction of the DGG model is calculated based on the following formula:


(3) $$p=\underset{t\to \infty }{\lim}s\left(t\right)={\left(1-\frac{\theta e^{x^{T}\beta }}{\alpha }\right)}^{-\frac{1}{\theta }}$$

The survival function of the 3- parameter Gompertz distribution with gamma frailty term is as follows:


(4) $$S\left(t\left|x\right.\right)={\left\{1+\theta \frac{e^{x^{T}\beta }}{\alpha \eta }\left(e^{\eta e^{\alpha T}}-e^{\eta }\right)\right\}}^{-\frac{1}{\theta }}$$

Here, α and η are shape parameters and belong to real values, θ>0 is the frailty term, and x^T^β=b_0_+b_1_x_1_+b_2_x_2_+…b_k_x_k_ in which β=(b_0_,b_1_,…, b_k_) indicates the coefficients vector and x^T^=(1,x_1_, x_2_,…, x_k_) indicates the covariates vector. When α<0, we have the defective gamma 3-parameter Gompertz (DGG3) model and θ∈R.

 The hazard function of the DGG3 model is as follows:


(5) $$h\left(t\left|x\right.\right)=e^{\alpha t+\eta e^{\alpha t}+x^{T}\beta }{\left\{1+\theta \frac{e^{x^{T}\beta }}{\alpha \eta }\left(e^{\eta e^{\alpha T}}-e^{\eta }\right)\right\}}^{-1}$$

The cure fraction of the DGG3 model is calculated based on the following formula:


(6) $$p=\underset{t\to \infty }{\lim}s\left(t\right)={\left(1-\frac{\theta e^{x^{T}\beta }}{\alpha \eta }\left(e^{\eta }-1\right)\right)}^{-\frac{1}{\theta }}$$

The backward selection method was used to find more useful predictors among all the predictors in the models. The backward selection is a method of fitting models with all candidate variables and testing the deletion of each variable using a chosen model fit criterion. In this method, the variable whose loss leads to the most insignificant deterioration of the model fit is deleted and this process is repeated until no further variables can be deleted from the model without any significant loss of fit. Selection aims to reduce the set of predictor variables to those that are necessary and account for nearly as much of the variance as is accounted for by the total set^30^. The best model was selected based on Akaike Information Criteria (AIC) and by comparison of the fitted survival curves with the Kaplan-Meier curves. The lower the AIC and the closer the fitted curves to the Kaplan-Meier curves, the better the model.

For better interpretability of the results, cut-off values were considered for the selected predictors. Two methods were used to find the cut-off values. The first method was using a receiver operating characteristics curve (ROC) and calculating the Youden index. The Youden index is calculated based on the following formula: J=sensitivity+specificity-1. In this formula, sensitivity is the ability of a test to correctly identify patients with recurrence, and specificity is the ability of a test to correctly identify people without recurrence. The Youden index value ranges from zero to one, and a value of one indicates that the test is perfect. The index is calculated for all points of a ROC curve, and the maximum value of the index is used as a criterion for selecting the optimum cut-off point. This method only considers the recurrence status^31,32^.

The second method was using the log-rank test which considers the survival times in addition to the recurrence status of the patients. It is a nonparametric test and is appropriate for usage in survival analysis^33^. Parameters of the models were estimated using the maximum likelihood estimation method. Maximum likelihood estimation is a method of estimating the parameters of a probability distribution by maximizing the likelihood function in a way that the observed data is most probable under the assumed statistical model. For this purpose, a code was written in R software (version 3.2.1) and the Broyden–Fletcher–Goldfarb–Shanno maximization method in the Optim package was used^34^. The codes are available upon request.

## Results

Table 1 summarizes the demographic and clinical information of 307 patients treated with CIN excisional surgery. The median and mean values of the survival time of patients were 60 and 75.17 months, respectively (95% CI: 72.54, 77.80). Moreover, the minimum and maximum follow-up times were 12 and 84 months, respectively. It is also noteworthy that recurrence was observed in 38 (12.4%) patients. The model containing the excised mass size and NLR variables had the lowest AIC among the fitted models; therefore, these two variables were selected to predict the cure rates among patients.

**Figure 2:**
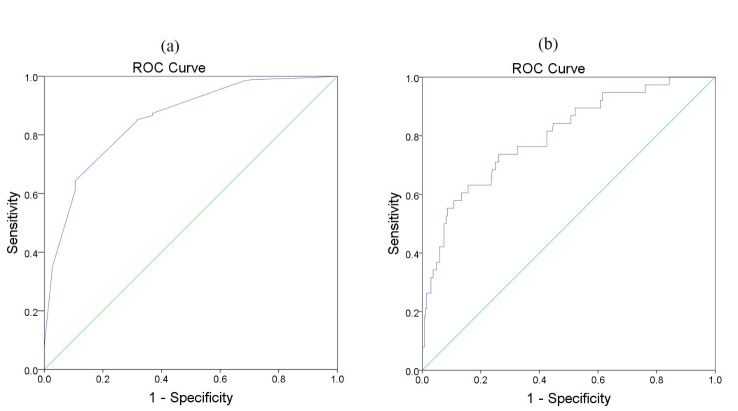
Receiver operating characteristics curves of (a) excised mass size, and (b) neutrophil-to-lymphocyte ratio for predicting recurrence

The area under the ROC curve (AUC) was remarkable for the diagnosis of recurrence based on the excised mass size predictor (AUC=0.852; 95% CI: 0.789, 0.916; *P*<0.001). The cut-off point of the excised mass size was obtained at 15 mm^2^ using the Youden index. The sensitivity and specificity values of the excised mass size cut-off point in the diagnosis of recurrence were 0.874; (95% CI: 0.678, 0.905) and 0.732; (95% CI: 0.653, 0.789), respectively (Figure 2.a). Furthermore, a cutting point of 15 mm^2^ was found for the excised mass size using the log-rank test (*P*<0.001). The AUC of the ROC curve for the diagnosis of recurrence based on the NLR predictor was remarkable (AUC=0.801; 95% CI: 0.723, 0.879; *P*<0.001).

The NLR cut-off point of 1.9 was obtained using the Youden index. The sensitivity and specificity values of the NLR cut-off point in the diagnosis of recurrence were 0.737 (95% CI: 0.569, 0.866) and 0.739 (95% CI: 0.682, 0.79), respectively (Figure 2.b). In addition, a cutting point of 1.9 was found for NLR using the log-rank test (*P*<0.001). The excised mass size was <15 mm^2^ in 58 (18.9%) patients and ≥15 mm^2^ in 249 (81.1%) patients. The recurrence rates in patients with low and high excised mass sizes were 41.4% and 5.6%, respectively. The NLR was <1.9 in 209 (68.1%) and ≥1.9 in 98 (31.9%) patients. The recurrence rates in patients with NLR of <1.9 and >1.9 were 4.8% and 28.6%, respectively.

Estimated values of the parameters of the DGG model are (α, θ, b_0_, b_1_, b_2_) = (-2.36, -0.51, -0.53, -1.92, 1.56) with standard errors of 0.23, 0.97, 0.26, 0.11, and 0.07 respectively. In the parameters vector, b_0_, b_1_, and b_2_ are the intercept, the coefficient of the excised mass size variable (*P*<0.001), and the coefficient of the NLR variable (*P*<0.001), respectively. The estimated values are substituted in formula 3 and the cure fractions are calculated for different categories of the covariates. It must be noted that the covariates x_1_=excised mass size and x_2_=NLR are considered as discrete and take zeros and ones for values lower and greater than the cut-off point values, respectively.

**Table 1:** Description and comparison of the demographic and hematologic factors of patients based on the NLR and the excised mass size levels

**Variables**	**Total (n=307)**		**NLR<1.9 and** **mass size>15** **(n=175)**		**NLR>1.9 and** **mass size>15** **(n=74)**		**NLR<1.9 and** **mass size<15** **(n=33)**		**NLR>1.9 and** **mass size<15** **(n=25)**		***P-*** **value**
**Continuous**	**Mean**	**SD**	**Mean**	**SD**	**Mean**	**SD**	**Mean**	**SD**	**Mean**	**SD**	
Age	40.36	9.14	40.57	9.59	40.62	8.43	40.76	8.54	37.6	8.70	0.479
Parity	2.74	1.91	2.79	1.09	2.61	1.94	2.94	1.69	2.522	1.53	0.758
MCV	84.71	8.50	84.92	7.62	84.63	7.61	83.64	14.65	84.81	5.98	0.888
HB	12.317	1.19	12.22	1.09	12.55	1.19	12.75	1.24	11.68	1.44	0.069
RBC	4.37	0.47	4.33	0.40	4.49	0.48	4.34	0.59	4.34	0.41	0.863
WBC	7263.84	1842.69	7106.29	1695.67	7224.32	1637.56	7096.97	1669.29	7304.00	1861.88	0.061
PLT	259.74	64.66	262.02	61.47	256.08	73.89	256.75	62.47	258.52	63.31	0.912
PLR	113.27	46.10	108.86	37.41	120.74	50.90	102.96	30.76	115.76	64.55	0.061
**Categorical**	**Number**	**Percent**	**Number**	**Percent**	**Number**	**Percent**	**Number**	**Percent**	**Number**	**Percent**	***P-*** **value**
Delivery Type											0.098
NVD	228	74.3	130	74.3	53	71.6	25	75.8	20	80	
CS	60	19.2	39	22.3	11	14.9	6	18.2	4	16	
None	19	6.5	6	3.4	10	13.5	2	6.1	1	4	
Cigarette											0.542
Yes	10	3.3	5	2.9	4	5.4	0	0	1	4	
No	297	96.7	170	97.1	70	94.6	33	100	24	96	
Treatment type											0.148
LEEP	236	76.9	128	73.1	60	81.1	25	75.8	23	92	
Conization	71	23.1	47	26.9	14	18.9	8	24.2	2	8	
Human Papilloma Virus											0.359
Positive	35	11.4	24	13.7	7	9.5	1	3	3	12	
Negative	9	2.9	5	2.9	1	1.4	1	3	2	8	
Unknown	263	85.7	146	83.4	66	89.2	31	93.9	20	80	
Margin											0.059
Involved	28	9.1	14	6.9	7	10.8	3	6.1	4	24	
Uninvolved	279	90.9	163	93.1	66	89.2	31	93.9	19	76	
Treatment cause											0.597
CIN I	46	14.9	22	12.6	14	19.6	6	17.2	4	15.4	
CIN II	204	66.5	117	67.2	47	65.2	25	71.4	15	57.7	
CIN III	57	18.6	35	20.2	11	15.2	4	11.4	7	25.9	

NLR: neutrophil-to-lymphocyte ratio, MCV: mean corpuscular volume, Hb: hemoglobin concentration, RBC: Red blood cell count, WBC: white blood cell count, PLT: platelet, PLR: platelet-to-lymphocyte ratio, NVD: natural vaginal delivery, CS: cesarean section, LEEP: Loop Electrosurgical Excision Procedure, CIN: cervical intraepithelial neoplasia

**Figure 3:**
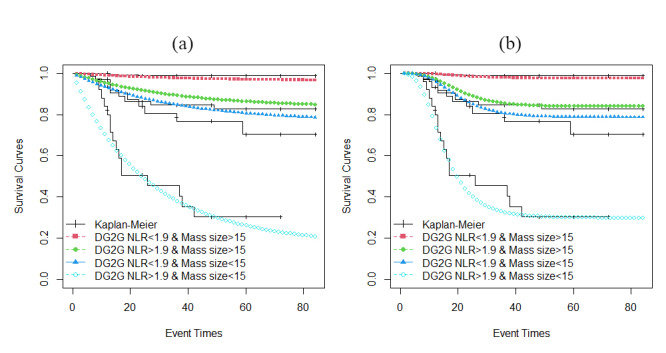
Comparison of the Kaplan-Meier curves with (a) defective gamma Gompertz and (b) defective gamma 3-parameters Gompertz models

Figure 3.a shows the comparison of the Kaplan-Meier curves with the curves of the fitted DGG model. The cure fractions of patients with the excised mass size of >15 mm^2^ and NLRs of <1.9 and >1.9 were estimated at 96% and 83%, respectively. Furthermore, cure fractions of patients with the excised mass size of < 15 mm^2^ and NLRs of <1.9 and >1.9 were estimated to be 76% and 16%, respectively.

Estimated values of the parameters of the DGG3 model are (α,θ,η,b_0_, b_1_,b_2_)=(-9.71, 0.82,-7.58, 2.97, -2.39, 2.05) with standard errors of 0.16, 0.42, 0.37, 0.05, 0.09, and 0.11, respectively. In the parameters vector, b_0_, b_1_, and b_2_ are the intercept, coefficient of the excised mass size variable (*P*<0.001), and coefficient of the NLR variable (*P*<0.001), respectively. The estimated values are substituted in formula 6 and the cure fractions are calculated for different categories of the covariates. As described above, the covariates are considered discrete. Figure 3.b shows the comparison of the Kaplan-Meier curves with the curves of the fitted DGG3 model.

The cure fraction among patients with the excised mass size of >15 mm^2^ and NLR <1.9 is estimated to be 98%, among patients with excised mass size >15 mm^2^ and NLR>1.9 is estimated to be 84%, among patients with excised mass size<15 mm2 and NLR<1.9 is estimated to be 79%, and among patients with excised mass size<15 mm2 and NLR>1.9 is estimated to be 30%.

The AICs of the DGG and DGG3 models are 123.1 and 105.3, respectively. Furthermore, according to Figure 3, the estimated curves of the DGG3 model were flattened close to Kaplan-Meier curves, while the curves of the DGG model are still decreasing. Hence, it can be said that the DGG3 model provides a better fit to the data.

Based on the survival curves of the DGG3 model (Figure 3.b) which was the best model based on AIC criteria, it can be said that patients with the excised mass size of >15 mm^2^ and NLR <1.9 are cured provided that they do not experience recurrence until 31 months after the treatment. Moreover, patients with the excised mass size of >15 mm^2^ and NLR of >1.9 are cured provided that they do not experience recurrence until 55 months after the treatment. In addition, patients with the excised mass size of <15 mm^2^ and NLR of <1.9 are cured provided that they do not experience recurrence until 52 months after the treatment. Finally, patients with the excised mass size of <15 mm^2^ and NLR of >1.9 are cured provided that they do not experience the recurrence until 59 months after the treatment. These time limits are based on the time (horizontal axis) where the fitted survival curves reach a plateau.

## Discussion

 In this study, it was found that the lower levels of NLR before surgery and an increase in the excised mass size lead to an increase in the cure rate from CIN recurrence among patients who underwent an excisional procedure for CIN. Cure rate models are important methods for the analysis of time-to-event data when there are risk-free individuals. It was common to use mixture models for cure rate modeling; however, recently, defective models are used for the analysis of data with cure fraction.

Defective models have the advantage of allowing a cure rate without requiring any extra parameters in the model and the proportion of the cured people is obtained by calculating the limit of the survival function and substituting the estimated parameters^28^. Moreover, the results of a study that compared the defective models with the mixtures model have indicated that the defective models fit better than the mixture models^28^. Presence of a frailty term in the model accounts for the unobserved heterogeneity and improves the fit of the model. The DGG3 model had a better fit on the data in comparison with the DGG model. Despite the existence of cured people in this dataset, the inverse Gaussian distribution could not estimate the cure fraction.

It was found that patients with lower NLR levels have more cure rates from CIN recurrence. Several studies on the effect of NLR, as a prognostic factor, on different cancers, such as colorectal, lung, and cervical cancers, revealed that patients with higher NLR levels prior to surgery had a shorter disease-free survival^27,35-37^. Misunuma et al.^38^ and Chun et al.^39^ also investigated the relationship between NLR and recurrence-free survival after the excisional procedure for the treatment of CIN. They found the same results but suggested a cut-off value of 2.1 for the NLR with sensitivity and specificity values of 0.571 and 0.745, respectively. The cut-off value of 1.9 for NLR found in this study had more sensitivity and specificity than that used by Chun et al.^39^.

It was found that the size of the excised tissue during excisional surgery had a direct relationship with the cure rate. Results of a similar study showed that a one-centimeter increase in the excised mass size reduced the hazard of recurrence by 68%. In the aforementioned study, it was also declared that due to the multifocal nature of CIN, removing larger tissues during surgery is accompanied by skip lesion and can decrease the chance of recurrence^40^. Nevertheless, the side effects, such as pregnancy-related complications, should be considered and a balance should be maintained between the two.

## Conclusions

 To prevent cancer and infertility, CIN patients must be identified and treated before the disease progresses and becomes invasive. Based on the findings, the NLR and excised mass size were the strongest predictive factors of the CIN recurrence. Removal of larger tissues during surgery, especially among patients with high NLR levels before the operation, can decrease the chance of recurrence after treatment. It should also be noted that using appropriate cure rate models is very important when there is a cure fraction in the data, otherwise, it may lead to incorrect estimates of the cure rates.

## Acknowledgments

 The authors would like to thank the reviewers and the editors for their comments which greatly improved this paper.

## Conflict of interests

 The authors declare that there are no conflicts of interest in this study.

## Funding

 This research is not financially supported.

## Highlights

- Neutrophil–to–lymphocyte ratio before surgery and excised mass size had significant impacts on cure rates among cervical intraepithelial neoplasia patients.
- Patients with a neutrophil–to–lymphocyte ratio of lower than 1.9 had a higher chance of getting cured.
- Patients with an excised mass larger than 15 mm^2^ had a higher chance of getting cured.
- An increase in excised mass size during surgery has a direct relationship with the cure rate especially among patients with a high neutrophil–to–lymphocyte ratio.
- When there is a cured fraction in data, it is important to use suitable cure models for the achievement of reliable results. Defective models are good choices for flexibly modeling the cure rate.
